# Systematic identification of miRNA-regulatory networks unveils their potential roles in sugarcane response to *Sorghum mosaic virus* infection

**DOI:** 10.1186/s12870-022-03641-6

**Published:** 2022-05-19

**Authors:** Yachun Su, Qiong Peng, Hui Ling, Chuihuai You, Qibin Wu, Liping Xu, Youxiong Que

**Affiliations:** 1 Key Laboratory of Sugarcane Biology and Genetic Breeding, Ministry of Agriculture and Rural Affairs, Fujian Agriculture and Forestry University, Fuzhou, 350002 Fujian China; 2 Fuzhou Institute of Agricultural Sciences, Fuzhou, 350018 Fujian China; 3 College of Agriculture, Yulin Normal University, Yulin, 537000 Guangxi, China; 4 College of Life Sciences, Fujian Agriculture and Forestry University, Fuzhou, 350002 Fujian China

**Keywords:** Sugarcane, *Sorghum mosaic virus*, miRNA, Target gene, Regulatory network

## Abstract

**Background:**

Sugarcane mosaic disease (SMD) is a major viral disease of sugarcane (*Saccharum* spp.) worldwide. *Sorghum mosaic virus* (SrMV) is the dominant pathogen of SMD in the sugarcane planting areas of China. There is no report on miRNAs and their regulatory networks in sugarcane response to SrMV infection.

**Results:**

In this study, small RNA sequencing (sRNA-seq) of samples from the leaves of SMD-susceptible variety ROC22 and -resistant variety FN39 infected by SrMV was performed. A total of 132 mature miRNAs (55 known miRNAs and 77 novel miRNAs) corresponding to 1,037 target genes were identified. After the SrMV attack, there were 30 differentially expressed miRNAs (17 up-regulated and 13 down-regulated) in FN39 and 19 in ROC22 (16 up-regulated and 3 down-regulated). Besides, there were 18 and 7 variety-specific differentially expressed miRNAs for FN39 and ROC22, respectively. KEGG enrichment analysis showed that the differentially expressed miRNAs targeted genes involved in several disease resistance-related pathways, such as mRNA surveillance, plant pathway interaction, sulfur metabolism, and regulation of autophagy. The reliability of sequencing data, and the expression patterns / regulation relationships between the selected differentially expressed miRNAs and their target genes in ROC22 and FN39 were confirmed by quantitative real-time PCR. A regulatory network diagram of differentially expressed miRNAs and their predicted target genes in sugarcane response to SrMV infection was sketched. In addition, precursor sequences of three candidate differentially expressed novel miRNAs (nov_3741, nov_22650 and nov_40875) were cloned from the ROC22 leaf infected by SrMV. Transient overexpression demonstrated that they could induce the accumulation of hydrogen peroxide and the expression level of hypersensitive response marker genes, salicylic acid-responsive genes and ethylene synthesis-depended genes in *Nicotiana benthamiana*. It is thus speculated that these three miRNAs may be involved in regulating the early immune response of sugarcane plants following SrMV infection.

**Conclusions:**

This study lays a foundation for revealing the miRNA regulation mechanism in the interaction of sugarcane and SrMV, and also provides a resource for miRNAs and their predicted target genes for SrMV resistance improvement in sugarcane.

**Supplementary Information:**

The online version contains supplementary material available at 10.1186/s12870-022-03641-6.

## Background

Sugarcane mosaic disease (SMD), an important viral disease of sugarcane worldwide, affects the yield and sugar content of sugarcane (*Saccharum* spp.) [1]. In China, SMD was first reported in 1968 and became one of the most serious diseases threatening the development of the sugarcane industry [2]. There are three main pathogens of SMD which belong to Potyviridae, including *Sugarcane mosaic virus* (SCMV), *Sorghum mosaic virus* (SrMV) and *Sugarcane streak mosaic virus* (SCSMV). As reported, these three pathogens can infect sugarcane either alone or together [1, 3]. The main pathogen of SMD in China is SrMV, followed by SCSMV which is mainly distributed in Yunnan and has the mixed infection ability [3, 4].

MicroRNA (miRNA), a type of single-stranded non-coding endogenous small RNA (sRNA) with a length of 20–25 nt, is cut from a single-stranded RNA precursor (pri-miRNA) with a length of 70–80 nt [5]. It can regulate gene expression and participate in plant disease resistance through complementary pairing with its target genes [5]. Xia et al. [6] found that 367 miRNAs were profiled in *Zea mays* and most of them were differentially expressed after SCMV infection. Niu et al. [7] reveals that miR159 targets the *P69* gene of *Turnip mosaic virus* (TuMV) and *HC-Pro* gene of *Tymovirus* (TYMV), and miR159-expressing transgenic *Arabidopsis thaliana* plants can resist infection from both viruses. Luan et al. [8] speculated that miR6027 and miR476b of *Lycopersicon esculentum* Mill may be involved in the regulation of silent plant immune response, thereby conferring tomato resistance to *Phytophthora infestans*. Boccara et al. [9] revealed that the resistance of miR472 mutant *Arabidopsis* plants to *Pseudomonas syringae* was significantly enhanced, while the transgenic plants overexpressing miR472 was more sensitive. It is interesting that miRNA is also related to virus-induced gene silencing [10]. Virus-mediated post-transcriptional silencing is one of the most important mechanisms by which plants resist viral infection, but some viruses can disrupt their antiviral defense mechanisms by interfering with plant miRNA expression [10]. For example, the post-transcriptional gene silencing suppressor AC4 encoded by *African cassava mosaic virus* (ACMV) can bind to miR159, interfere with its regulation of target genes, and affect the growth and development of *Arabidopsis* [11].

In sugarcane, a SCSMV miRNA (miR16) and its 19 target genes were identified by Viswanathan et al. [12]. Further, they indicated that miR16 plays a regulatory role by cleaving target genes to establish a virus living environment and resist the host defense response [12]. Ling et al. [13] demonstrated that protein phosphatase 2A (*PP2A*) gene and miR159 constituted the best single sugarcane reference gene / miRNA under SrMV infection. Till now, the systematic identification of miRNAs and their regulatory networks in sugarcane response to SrMV infection has not yet been reported.

In the present study, the SMD-resistant variety FN39 and -susceptible variety ROC22 were used to construct a miRNA library of sugarcane responding to SrMV infection by small RNA sequencing (sRNA-seq). According to the changes in miRNA expression abundance, the differentially expressed miRNAs and their corresponding target genes induced by SrMV infection were identified. Quantitative real-time PCR (qRT-PCR) was then used to detect the expression level of 12 selected differentially expressed miRNAs and their 15 predicted target genes in ROC22 and FN39 infected by SrMV. Moreover, precursor sequences of three candidate differentially expressed novel miRNAs (nov_3741, nov_22650 and nov_40875) were cloned from sugarcane and their preliminary roles in immune responses were verified by transient overexpression in *Nicotiana benthamiana*. This study helps to reveal the molecular mechanism of miRNAs regulating sugarcane disease resistance and susceptibility under SrMV infection and provides a reference for sugarcane disease resistance breeding.

## Results

### Categories of sRNAs, and size and first base distribution of miRNAs in sugarcane infected by SrMV

**Fig. 1:**
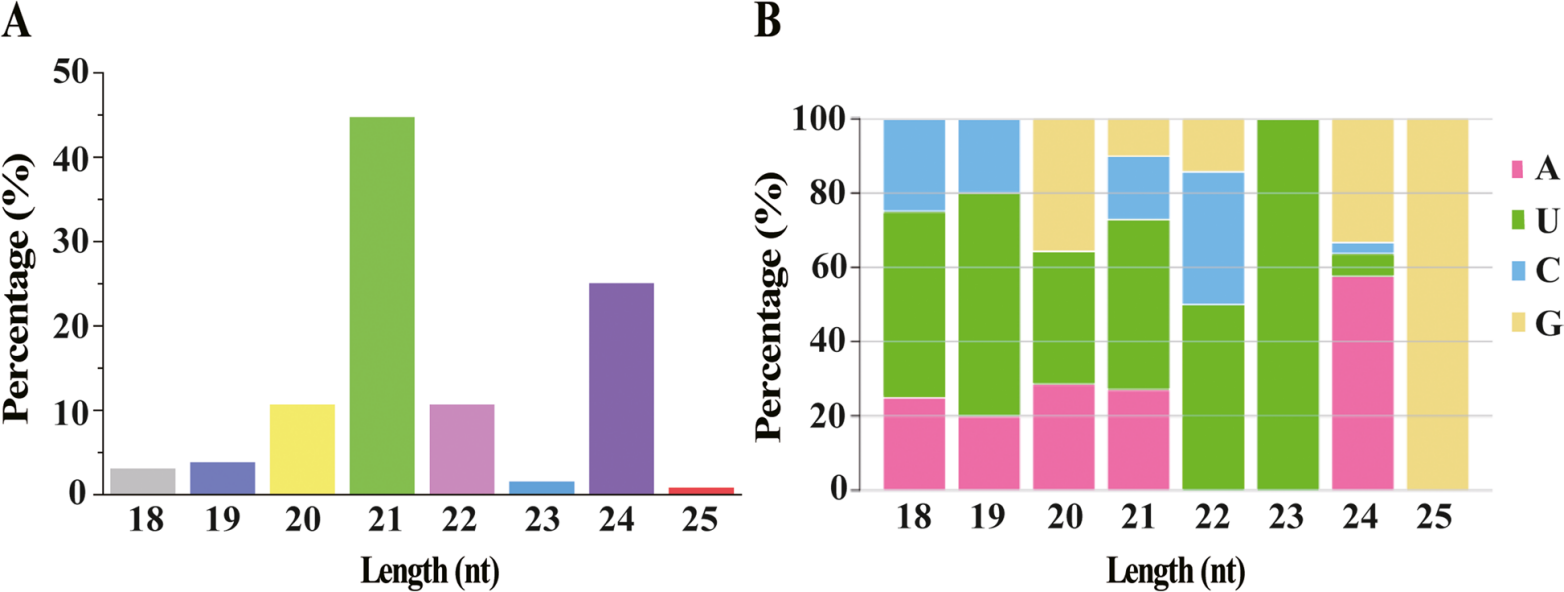
Relative frequency of length (**A**) and 5’ terminal nucleotide (**B**) of miRNA sequences in libraries prepared from sugarcane varieties ROC22 and FN39 infected by *Sorghum mosaic virus*. The *y*-axis shows the percentage of reads; the *x*-axis shows the sequence size, from 18 to 25 nt

A total of 174.71 M clean reads was generated from the 12 sugarcane samples. The clean data of each sample was above 12.30 M and accounted for more than 60% of the raw reads, and the average value of Q30 was above 91% (Table S1), indicating that the sequencing data meets the subsequent analysis. The classification and annotation results showed that the clean reads can be classified into ribosomal RNA (rRNA), small nucleolar RNA (snoRNA), transfer RNA (tRNA), repeat sequence (Repbase) and unannotated reads (Table S2). After comparison, 132 mature miRNAs including 38 miRNA families were obtained, of which 55 were known miRNAs and 77 were novel miRNAs. To exclude the influence of SrMV miRNAs, all the identified miRNAs were compared with the genome of SrMV. The align results showed that the sequences of these 132 miRNAs did not match the SrMV genome sequence. The length of the identified miRNAs was mainly 21 nt (44.70%) and 24 nt (25%) (Fig. 1A). Figure 1B showed that the 5' terminal nucleotide of 21 nt miRNAs was mainly biased towards U, while that of the 24 nt miRNAs was mainly biased towards A, which was consistent with the previous reports that the 5' terminal nucleotide of plant miRNAs has a certain preference for A/U base [14, 15].

### The differentially expressed miRNAs in the sugarcane SMD resistant variety were more than that in the susceptible variety infected by SrMV

**Fig. 2:**
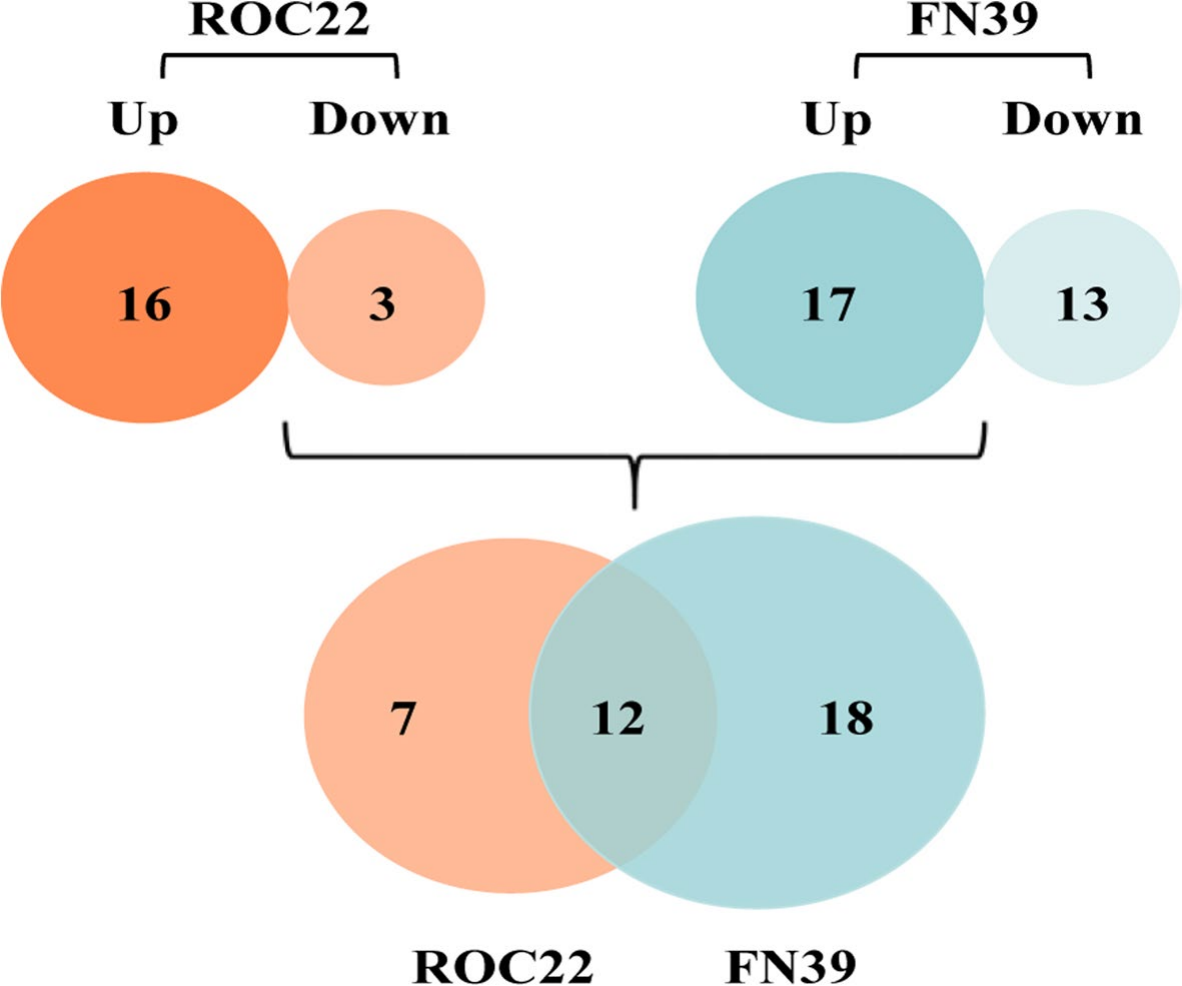
The differentially expressed miRNAs in susceptible ROC22 and resistant FN39 infected by *Sorghum mosaic virus*. Up and down respectively represent up- and down-regulated differentially expressed miRNAs

Among the 132 miRNAs, 37 differentially expressed miRNAs between the control and treatment groups of two sugarcane varieties were screened. Interestingly, after SrMV infection, the number of differentially expressed miRNAs in the resistant variety FN39 (30) was more than that of the susceptible variety ROC22 (19). There were 17 up- and 13 down-regulated differentially expressed miRNAs in FN39, including 12 known miRNAs and 18 novel miRNAs (Fig. 2 and Table S3). In ROC22, there were 16 up- and 3 down-regulated differentially expressed miRNAs, including 8 known and 11 novel miRNAs. The number of common differentially expressed miRNAs between ROC22 and FN39 was 12, while the number of variety-specific differentially expressed miRNAs was 7 and 18 for ROC22 and FN39, respectively.

### Targets of miRNA prediction

**Fig. 3:**
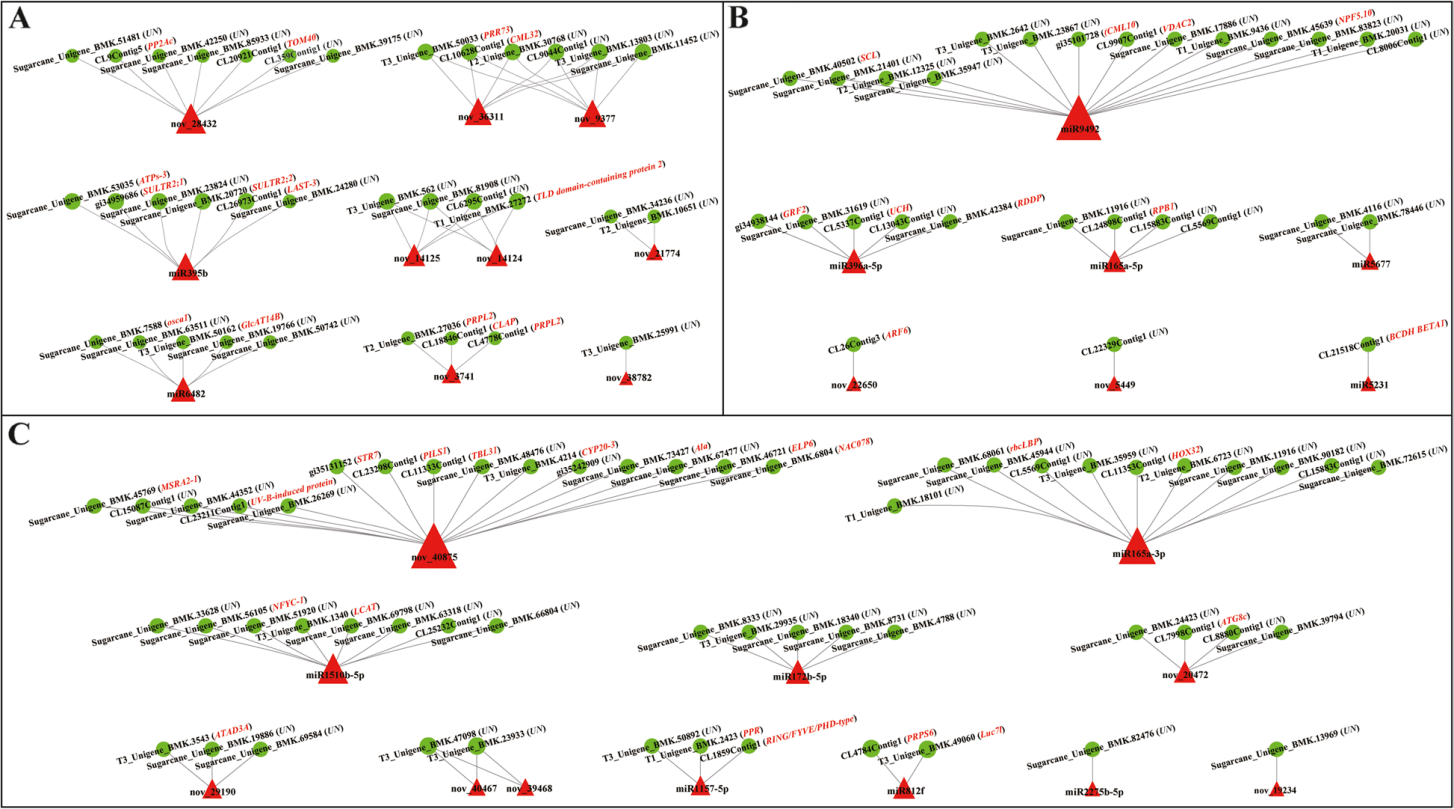
Networks showing the interaction between differentially expressed miRNAs and their corresponding predicted target genes. **A** The common differentially expressed miRNAs in both ROC22 and FN39. **B** The specific differentially expressed miRNAs in ROC22. **C** The specific differentially expressed miRNAs in FN39. ROC22 and FN39 respectively represent sugarcane mosaic disease-susceptible and -resistant varieties. Red triangles represent differentially expressed miRNAs, and green circles represent target genes. UN means a hypothetical protein with unknown function. The description of the annotated target gene follows its gene ID with a red font. *PP2Ac*, phosphatase 2A-3 catalytic subunit-like; *TOM40*, translocase of the outer mitochondrial membrane 40; *PRR73*, pseudo-response regulator 73; *CML32*, calcium-binding protein 32; *ATPs-3*, ATP-sulfurylase 3; *SULTR2;1*, sulfate transporter 2;1; *SULTR2;2*, sulfate transporter 2;2; *LAST-3*, low affinity sulfate transporter 3-like; *osca1*, reduced hyperosmolality-induced Ca^2+^ increase 1; *GlcAT14B*, beta-glucuronosyltransferase 14B; *PRPL2*, 50S chloroplastic ribosomal protein L2; *CLAP*, clathrin assembly protein; *SCL*, scarecrow-like protein; *CML10*, calmodulin-like protein 10; *VDAC2*, voltage-dependent anion channel 2; *NPF5.10*, protein NRT1/PTR family 5.10; *GRF2*, growth-regulating factor 2; *UCH*, ubiquitin carboxyl-terminal hydrolase; *RDDP*, RNA dependent DNA polymerase; *RPB1*, DNA-directed RNA polymerase V subunit 1; *ARF6*, auxin response factor 6; *BCDH BETA1*, branched-chain alpha-keto acid decarboxylase E1 beta subunit; *MSRA2-1*, peptide methionine sulfoxide reductase A2-1; *STR7*, rhodanese-like domain-containing protein 7; *PILS1*, pin-likes 1; *TBL31*, protein trichome birefringence-like 31; *CYP20-3*, peptidyl-prolyl cis–trans isomerase CYP20-3; *Ala*, alanine–tRNA ligase, chloroplastic; *ELP6*, elongator complex protein 6; *NAC078*, NAC domain-containing protein 78; *rbcLBP*, Rubisco large subunit-binding protein; *HOX32*, homeobox-leucine zipper protein; *NFYC-1*, nuclear transcription factor Y subunit C-1; *LCAT*, lecithin-cholesterol acyltransferase-like 1; *ATG8c*, autophagy-related protein 8C-like; ATAD3A, ATPase family AAA domain-containing protein 3-A; *PPR*, pentatricopeptide repeat-containing protein; *RING/FYVE/PHD-type*, RING/FYVE/PHD zinc finger superfamily protein; *PRPS6*, 30S chloroplastic ribosomal protein S6; and *Luc7l*, putative RNA-binding protein Luc7-like 1

Based on our previous transcriptome data of sugarcane [16, 17], a total of 1,037 target genes of 132 identified miRNAs were predicted. After comparing the predicted target gene sequences with Non-Redundant Protein Sequence Database (NR), Swiss-Prot, Gene Ontology (GO), Clusters of Orthologous Groups (COG), Kyoto Encyclopedia of Genes and Genomes (KEGG), euKaryotic Orthologous Groups (KOG) and Pfam database by BLAST software, 650, 388, 516, 174, 114, 313 and 461 target genes were respectively annotated, and the total number of annotated target genes was 653 (Table S4). Among the predicted target genes corresponding to 37 differentially expressed miRNAs, 14 genes were annotated corresponding to the common 14 differentially expressed miRNAs in both ROC22 and FN39 (Fig. 3A and Table S5). In addition, 10 and 19 genes were annotated corresponding to the specific differentially expressed miRNAs in ROC22 and FN39, respectively (Figs. 3B, C and Table S5). Most of these target genes were related to plant stress responses, e.g. ATP-sulfurylase 3 (*ATPs-3*) was a key catalytic enzyme in the plant thiometabolic pathway in response to plant sulfur deficiency stress [18]; autophagy-related protein 8C-like (*ATG8c*) was involved in the plant autophagy regulatory pathway, which was closely related to programmed cell death in allergic reactions [19]. It is important to note that each miRNA had at least one predicted target gene, and the same predicted target gene may be regulated by multiple miRNAs. For example, miR395b was predicted to target *ATPs-3* and low affinity sulfate transporter 3-like (*LAST-3*) genes, while calmodulin-like protein 32 (*CML32*) was assumed to be targeted by nov_36311 and nov_9377.

### GO and KEGG enrichment analysis of target genes

**Fig. 4:**
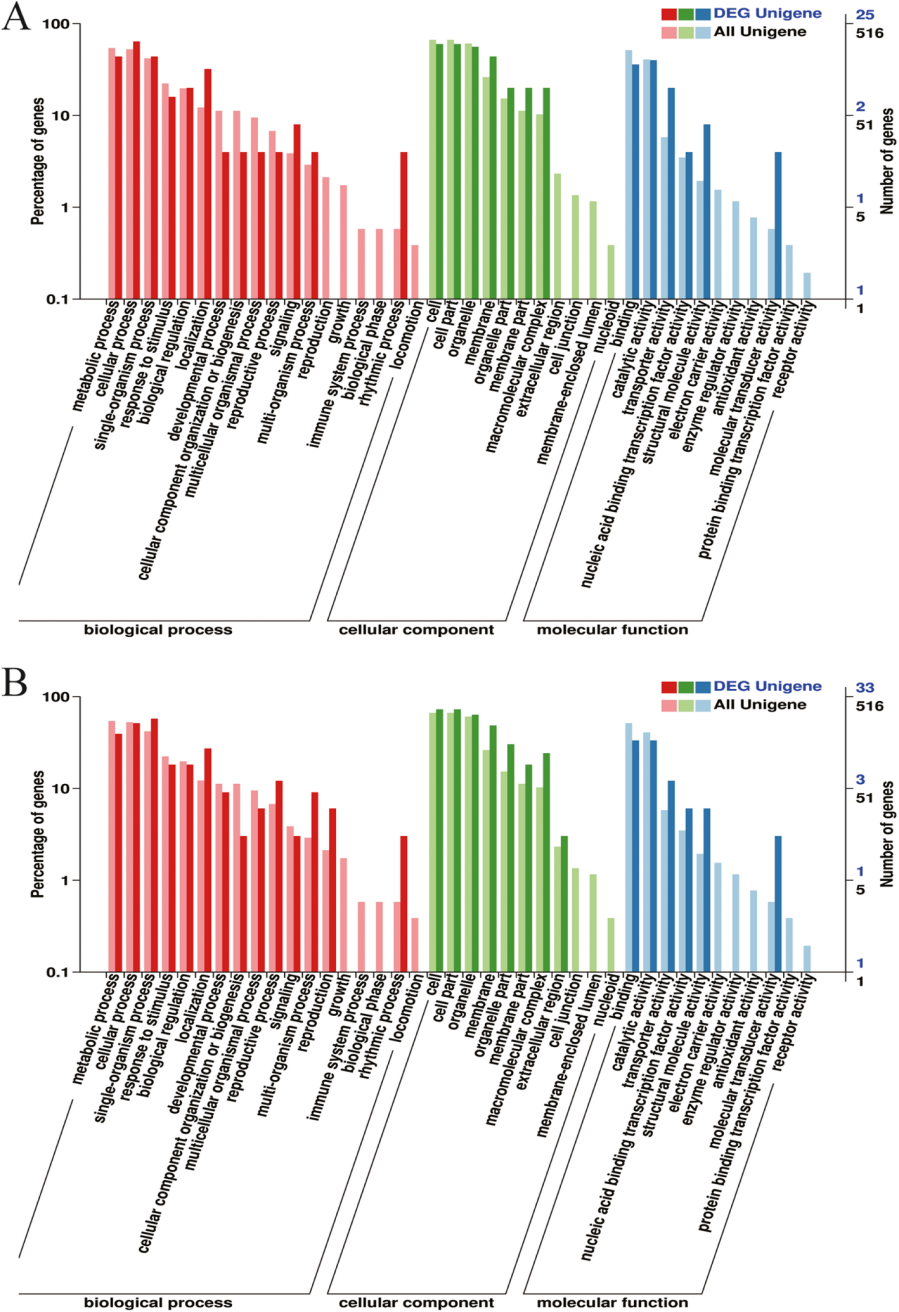
Gene Ontology (GO) enrichment analysis for the predicted target genes corresponding to differentially expressed miRNAs in susceptible ROC22 (**A**) and resistant FN39 (**B**) infected by *Sorghum mosaic virus*. The GO terms were categorized according to biological process, cellular component and molecular function. The left ordinate represents the percentage of predicted target genes, the right ordinate represents the number of predicted target genes, and the abscissa is the 40 GO function classification items

The results of GO enrichment analysis showed that 516 predicted target genes of miRNAs in the two sugarcane varieties involved in 40 GO terms. It should be pointed out that, for ROC22, 25 predicted target genes corresponding to 19 differentially expressed miRNAs participated in 26 GO terms, while for FN39, 33 predicted target genes corresponding to 30 differentially expressed miRNAs related to 28 GO terms. They were mainly enriched in cellular component, catalytic activity, metabolic process, cellular process and single-organism process (Fig. 4). KEGG enrichment analysis showed that 7 predicted target genes corresponding to 8 differentially expressed miRNAs were enriched in 9 pathways, including mRNA surveillance pathway, valine, leucine and isoleucine degradation, plant-pathogen interaction, aminoacyl-tRNA biosynthesis, regulation of autophagy, glycerophospholipid metabolism, selenocompound metabolism, sulfur metabolism and purine metabolism (Table 1), suggesting that these pathways and their related genes may play a role in ROC22 and FN39 response to SrMV infection.

**Table 1:** KEGG enrichment analysis of the predicted target genes corresponding to differentially expressed miRNAs in susceptible ROC22 and resistant FN39 infected by *Sorghum mosaic virus*

Variety	Pathway	Pathway ID	Enrichment factor	Q-value	Predicted target gene ID	miRNA ID
ROC22	mRNA surveillance pathway	ko03015	19.00	0.3158	CL9Contig5	unconservative_Sugarcane_Unigene_BMK.66937_28432
	Valine, leucine and isoleucine degradation	ko00280	19.00	0.3158	CL21518Contig1	conservative_CL21518Contig1_10252
	Selenocompound metabolism	ko00450	19.00	0.3158	Sugarcane_Unigene_BMK.53035	conservative_Sugarcane_Unigene_BMK.11814_18991; conservative_Sugarcane_Unigene_BMK.23824_20400
	Sulfur metabolism	ko00920	6.33	0.9098		
	Purine metabolism	ko00230	2.71	1.0000		
	Plant-pathogen interaction	ko04626	6.33	0.9098	gi35101728	conservative_CL26672Contig1_15829
FN39	Aminoacyl-tRNA biosynthesis	ko00970	15.20	0.4605	Sugarcane_Unigene_BMK.73427	unconservative_T3_Unigene_BMK.49787_40875
	Regulation of autophagy	ko04140	15.20	0.4605	CL7998Contig1	unconservative_Sugarcane_Unigene_BMK.24423_20472
	mRNA surveillance pathway	ko03015	15.20	0.4605	CL9Contig5	unconservative_Sugarcane_Unigene_BMK.66937_28432
	Selenocompound metabolism	ko00450	15.20	0.4605	Sugarcane_Unigene_BMK.53035	conservative_Sugarcane_Unigene_BMK.11814_18991; conservative_Sugarcane_Unigene_BMK.23824_20400
	Sulfur metabolism	ko00920	5.07	1.0000		
	Purine metabolism	ko00230	2.17	1.0000		
	Glycerophospholipid metabolism	ko00564	5.07	1.0000	T3_Unigene_BMK.1340	conservative_T1_Unigene_BMK.40479_34605

KEGG enrichment analysis was referred to the report of Kanehisa et al. [20]. Conservative and unconservative IDs represent known and novel miRNAs, respectively

### Clustering of the expression pattern of differentially expressed miRNAs

**Fig. 5:**
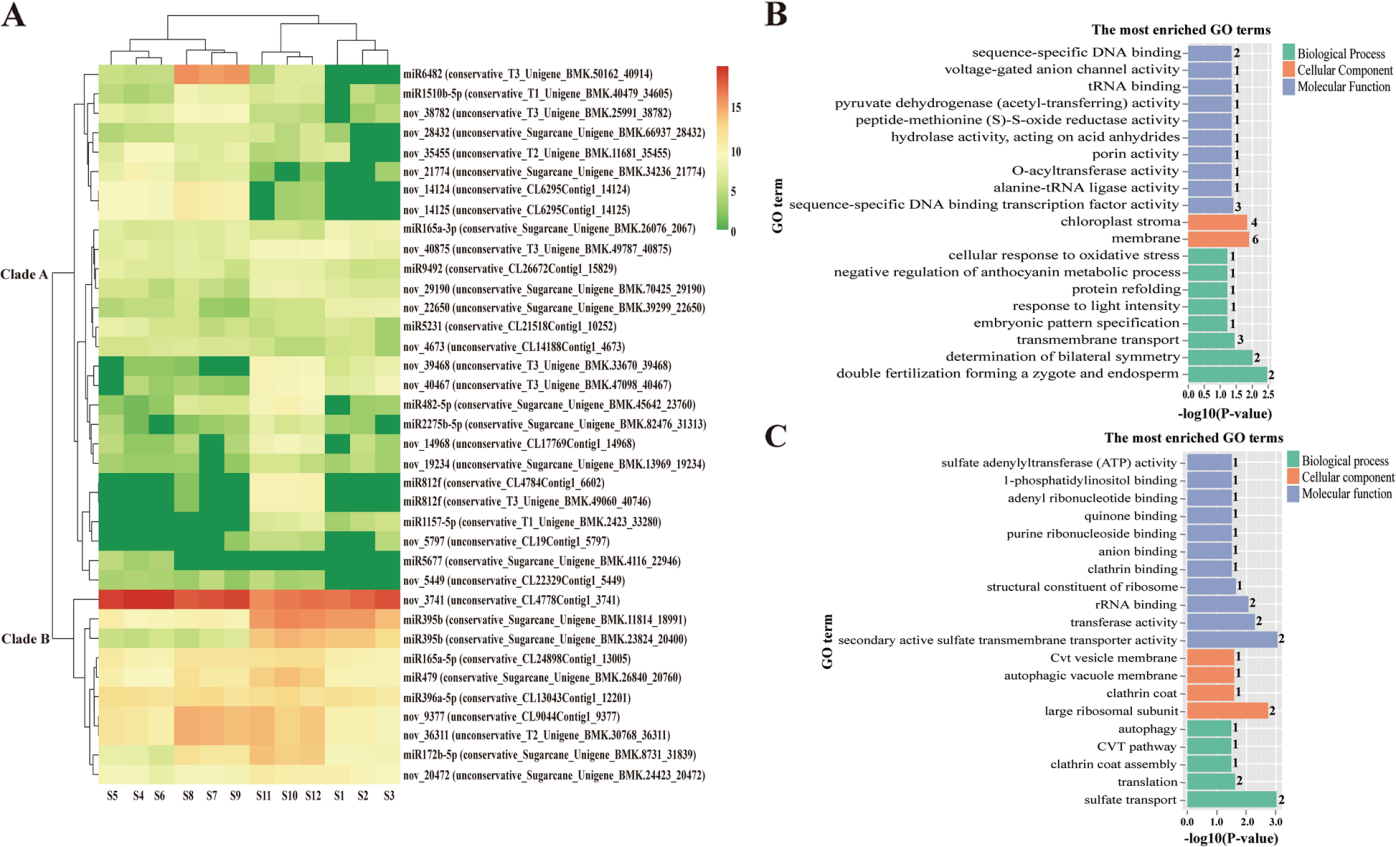
The expression clustering result of the 37 differentially expressed miRNAs and Go enrichment analysis of their predicted target genes in susceptible ROC22 and resistant FN39 infected by *Sorghum mosaic virus* (SrMV). **A** The expression clustering result of the 37 differentially expressed miRNAs. Each row represents a single miRNA, and each column represents a miRNA library. S1-S3 represent the healthy leaf samples of ROC22; S4-S6 represent the leaf samples of ROC22 infected by SrMV; S7-S9 represent the healthy leaf samples of FN39; S10-S12 represent the leaf samples of FN39 infected by SrMV. Conservative and unconservative IDs represent known and novel miRNAs, respectively. Clade B with red color indicates a high abundance of miRNA, and clade A with green color for the opposite situation. **B and C** Go enrichment analysis of the predicted target genes of differentially expressed miRNAs in clade A and clade B. The number of target genes were shown on the right of Go terms

Hierarchical clustering result showed that the expression patterns of the 37 differentially expressed miRNAs in 12 sRNA-seq samples were clustered in two clades (Fig. 5A). Among them, the transcript abundance of 27 differentially expressed miRNAs in clade A was relatively low (green color), while 10 in clade B was high (red color). The predicted target genes of miRNAs in clade A were mainly enrichmented in the GO terms of membrane, chloroplast stroma, sequence-specific DNA binding, sequence-specific DNA binding transcription factor activity, transmembrane transport, determination of bilateral symmetry, double fertilization forming a zygote and endosperm (Fig. 5B). In clade B, the miRNAs targeted genes mainly enrichmented in the GO terms of rRNA binding, transferase activity, secondary active sulfate transmembrane transporter activity, large ribosomal subunit, translation, and sulfate transport (Fig. 5C). These results indicated that miRNAs with different expression abundances may exhibit different roles in regulation of their target genes involved in various biological processes during the interaction between sugarcane and SrMV.

### Validation of miRNAs by qRT-PCR analysis

**Fig. 6:**
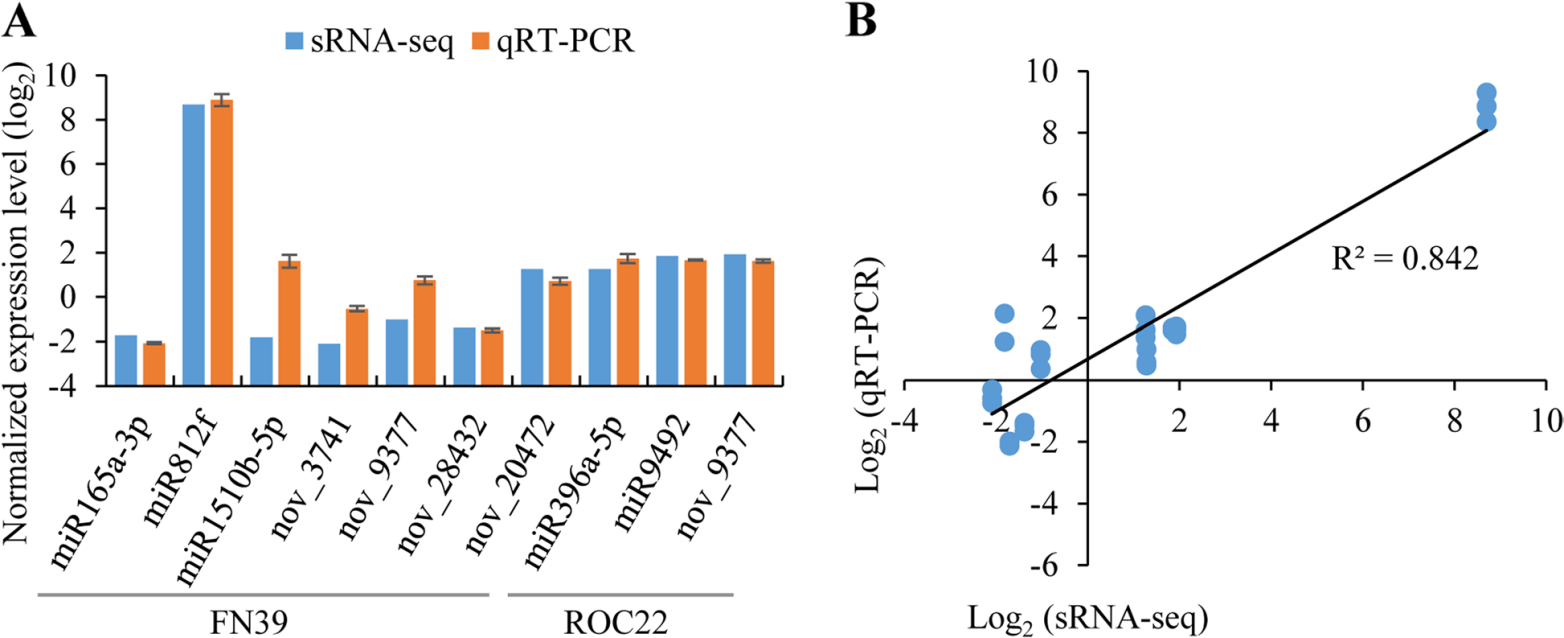
qRT-PCR analysis of miRNAs (**A**) and correlation of qRT-PCR (log_2_ scale) and sequencing data of these miRNAs (**B**). FN39 and ROC22 were sugarcane mosaic disease-resistant and -susceptible varieties, respectively. Using miR159 as an internal reference gene. Each bar represents the mean values of three replicates ± standard error (*n* = 3). The correlation analysis is performed by scatter plot, and the *y*-axis and *x*-axis in Fig. 6B respectively show log_2_ ratio of selected miRNAs expression in SrMV-infected samples versus virus-free samples via qRT-PCR and sRNA-seq

To verify the reliability of the sequencing results, the expression level of nine differentially expressed miRNAs (i.e. miR165a-3p, miR812f, miR1510b-5p, nov_3741, nov_9377, nov_28432, nov_20472, miR396a-5p and miR9492) were randomly selected for the qRT-PCR test (Fig. 6A). The transcript levels of selected miRNAs via qRT-PCR analysis (log_2_ scale) had a high correlation (R^2^ = 0.842) with that of the sRNA-seq data using the same samples (Fig. 6B).

### miRNAs and their target genes showed diverse expression patterns in ROC22 and FN39 infected by SrMV

**Fig. 7:**
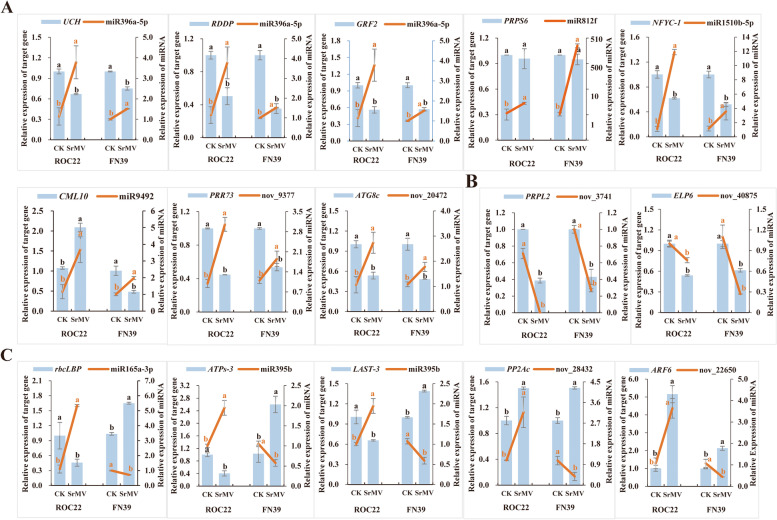
Expression patterns of the randomly selected 12 miRNAs (line charts) and their 15 target genes (bar charts) in susceptible ROC22 and resistant FN39 infected by *Sorghum mosaic virus* through qRT-PCR analysis. **A** The miRNAs and their target genes were all up-regulated in both varieties. **B** The miRNAs and their target genes were all down-regulated in both varieties. **C** The miRNAs and their target genes showed inconsistent expression patterns in susceptible and resistant varieties. CK and SrMV represent the virus-free sample (control group) and SrMV-infected plants with mosaic symptoms (treatment group), respectively. The right and left *y* axis represent the relative expression of miRNA and its predicted target gene, respectively. Each bar represents the mean values of three replicates ± standard error (*n* = 3). Different lowercase letters indicate a significant difference, as determined by the least-significant difference test (*p*-value < 0.05). *rbcLBP*, Rubisco large subunit-binding protein; *UCH*, ubiquitin carboxyl-terminal hydrolase; *RDDP*, RNA dependent DNA polymerase; *GRF2*, growth-regulating factor 2; *NFYC-1*, nuclear transcription factor Y subunit C-1; *CML10*, calmodulin-like protein 10; *PRPL2*, 50S chloroplastic ribosomal protein L2; *ARF6*, auxin response factor 6; *PP2Ac*, phosphatase 2A-3 catalytic subunit-like; *ATG8c*, autophagy-related protein 8C-like; *ELP6*, elongator complex protein 6; *PRR73*, pseudo-response regulator 73; *ATPs-3*, ATP-sulfurylase 3; *LAST-3*, low affinity sulfate transporter 3-like; and *PRPS6*, 30S chloroplastic ribosomal protein S6

The expression level of 12 randomly selected differentially expressed miRNAs and their corresponding 15 predicted target genes were analyzed in SMD-susceptible ROC22 and -resistant FN39 before and after infection by SrMV (Fig. 7). The results showed that the expression patterns of these miRNAs in the two sugarcane varieties were divided into two types, consistent and inconsistent.

Regarding the 8 miRNAs with consistent expression patterns in the SMD-susceptible and -resistant varieties, 6 miRNAs, miR396a-5p, miR812f, miR1510b-5p, miR9492, nov_9377 and nov_20472, were significantly up-regulated in both varieties (Fig. 7A). In ROC22, miR1510b-5p had the highest expression level, which was 10.81-times that of the control. Interestingly, the expression level of miR812f in FN39 was 476.33-times that of the control. The other two miRNAs (nov_3741 and nov_40875) were significantly down-regulated in the two varieties, of which the expression level of nov_3741 in ROC22 was 2.81 × 10^–4^-times that of the control (Fig. 7B). In addition, among these 8 miRNAs, the expression patterns of miR396a-5p, miR812f, miR1510b-5p, nov_9377 and nov_20472 were opposite to that of their target genes ubiquitin carboxyl-terminal hydrolase (*UCH*), 30S plastid ribosomal protein S6 (*PRPS6*), nuclear transcription factor Y subunit C-1 (*NFYC-1*), pseudo-response regulator 73 (*PRR73*) and *ATG8c*, suggesting that there may be a negative regulation relationship between these 5 miRNAs and their corresponding target genes. For example, miR9492 and its target gene *CML10* may exhibit a positive regulatory relationship in ROC22, however there was a negative regulatory relationship in FN39. It is worth noting that nov_3741 and nov_40875 may both play a positive regulatory role in their target genes 50S chloroplastic ribosomal protein L2 (*PRPL2*) and elongator complex protein 6 (*ELP6*) in the two varieties, whose expression level was all down-regulated.

As for the other 4 miRNAs, miR165a-3p, miR395b, nov_28432 and nov_22650, with inconsistent expression patterns in SMD-susceptible and -resistant varieties, were all significantly up-regulated in ROC22, but significantly down-regulated in FN39 (Fig. 7C). Besides, the expression level of miR165a-3p in ROC22 was the highest, which was 4.84-times that of the control, while there was little difference in the expression level (0.35–0.72-times) of the four miRNAs in FN39. It is thus speculated that miR165a-3p and miR395b may negatively regulate their target genes Rubisco large subunit-binding protein (*rbcLBP*) and *ATPs-3*/*LAST-3* in both varieties, while nov_22650 and nov_28432 may play a positive regulatory role in their target genes auxin response factor 6 (*ARF6*) and phosphatase 2A-3 catalytic subunit-like (*PP2Ac*) in ROC22 but a negative regulatory mode in FN39.

### Preliminary observation on immune response of three candidate sugarcane miRNAs by transient overexpression

**Fig. 8:**
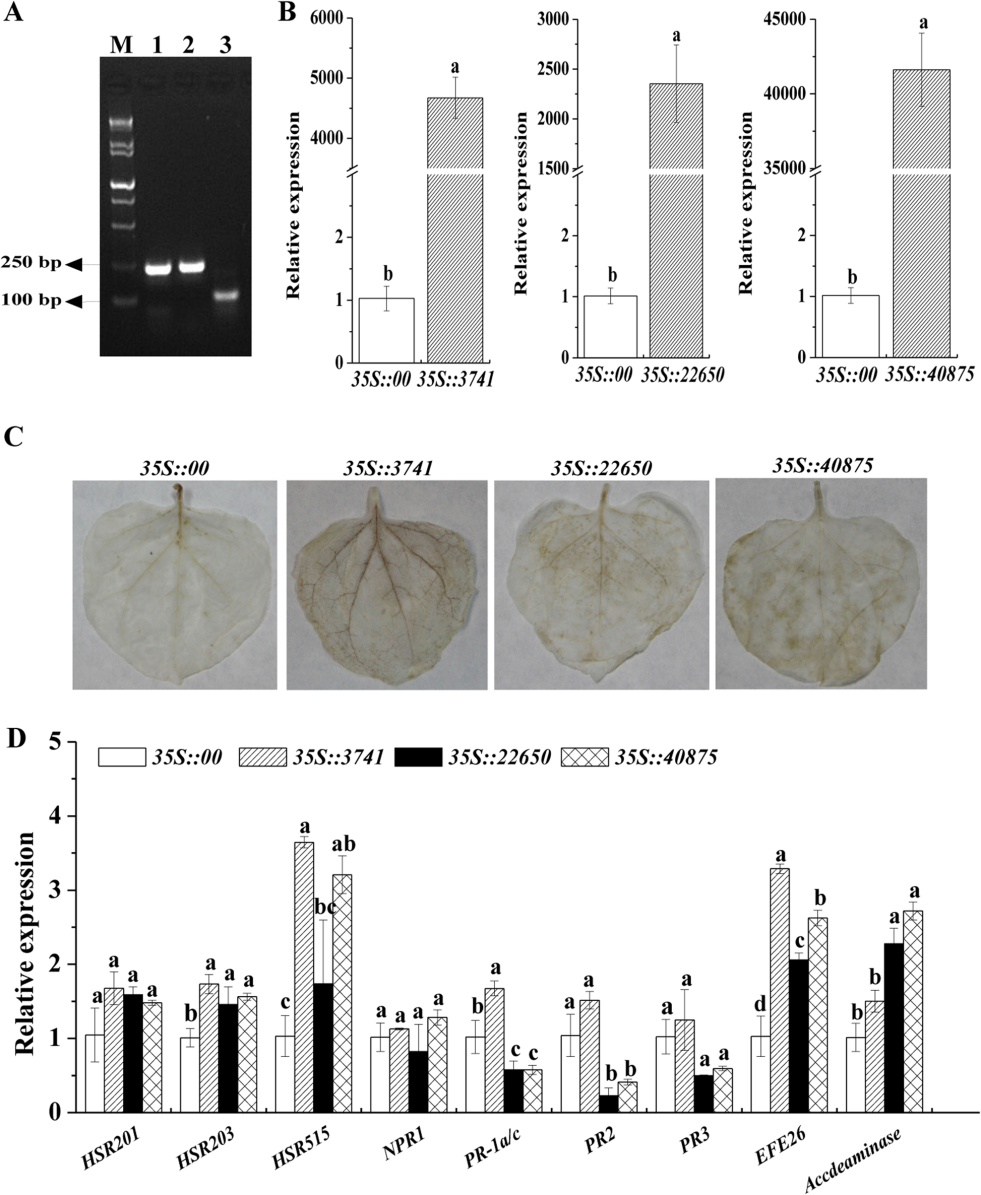
Analysis of immune effects induced by transient overexpression of three candidate sugarcane miRNAs in *Nicotiana benthamiana*. **A** PCR products of the three candidate sugarcane pre-miRNAs detected by electrophoresis in the same gel. M, DNA marker 15,000 + 2,000 bp; 1, pre-nov_3741; 2, pre-nov_22650; and 3, pre_nov_40875. **B** The transcript of three candidate miRNAs in *N. benthamiana* leaves at 2 d after agroinfiltration by qRT-PCR analysis. **C** DAB staining in *N. benthamiana* leaves infiltrated by *Agrobacterium tumefaciens* for 2 d. **D** The transcript of the immune-related marker genes at 2 d after agroinfiltration by qRT-PCR analysis, including the hypersensitive response marker genes *HSR201*, *HSR203* and *HSR515*, the salicylic acid-responsive genes *NPR1*, *PR-1a/c*, *PR2* and *PR3*, and the ethylene synthesis-depended genes *EFE26* and *Accdeaminase*. *EF1-α* was used to normalize the transcript level. All data points are the means ± SE (*n* = 3). Different lowercase letters indicate a significant difference (*p*-value < 0.05). *35S::00* represents the *A. tumefaciens* strains carrying the empty vector pCAMBIA1301. *35S::3741*, *35S::22650* and *35S::40875* represent the *A. tumefaciens* strains carrying the recombinant vector pCAMBIA1301-nov_3741, pCAMBIA1301-nov_22650 and pCAMBIA1301-nov_40875, respectively

To assess the functions of sugarcane miRNAs, three candidate differentially expressed novel miRNAs (nov_3741, nov_22650 and nov_40875) of the above six tested novel miRNAs, whose predicted target genes (*PRPL2*, *ARF6* and *ELP6*) may be involved in chloroplast function regulation, auxin and ABA signaling pathways, were cloned and identified. The precursor sequences of nov_3741, nov_22650 and nov_40875 with lengths of 244 bp, 245 bp and 116 bp were isolated from ROC22 leaf infected by SrMV (Fig. 8A and Fig. S2). After transient overexpression of nov_3741, nov_22650 and nov_40875, the hydrogen peroxide (H_2_O_2_) content and the expression level of tobacco immune-related marker genes in *N. benthamiana* leaves were analyzed (Figs. 8B, C, D). The results showed that the transcription level of three miRNAs in *N. benthamiana* leaves was significantly increased after 2 days of injection (Fig. 8B). The 3,3'-diaminobenzidine solution (DAB) staining colors of the leaves overexpressed *35S::3741*, *35S::22650* and *35S::40875* were darker than those of the control (*35S::00*) (Fig. 8C), indicating the accumulation of H_2_O_2_ content and the exhibition of allergic reaction. In addition, the expression level of hypersensitive response (HR) marker genes (*HSR203* and *HSR515*), salicylic acid (SA) pathway-related gene (*PR-1a/c*) and ethylene (ET) synthesis-depended gene (*EFE26*) was increased in *35S::3741* leaves (Fig. 8D). Whereas in *35S::22650* and *35S::40875* leaves, the expression level of HR marker genes (*HSR203* and *HSR515*) and ET synthesis-depended genes (*EFE26* and *Accdeaminase*) was increased, and that of the SA-responsive genes (*PR-1a/c* and *PR2*) was decreased (Fig. 8D). These results suggest that nov_3741, nov_22650 and nov_40875 may be involved in regulating the transcription levels of RNA transcripts to confer the early immune response to plants.

## Discussion

In this study, 132 sugarcane miRNAs with a main length of 21 nt and 24 nt were identified by sRNA-seq (Fig. 1), which was consistent with the reports of Baulcombe [21] and Jin et al. [22]. The mature miRNA is cleaved by the dicer enzyme with a specific restriction site. Researches have shown that the preferred base of mature miRNAs is mainly A/U, and the miRNAs with the 5’ terminal nucleotide as A/U can specifically bind to AGO4/AGO1, respectively [14, 15]. The 5' terminal nucleotide of the sugarcane 21 nt miRNAs was mainly biased towards U, while that of the 24 nt miRNAs was A (Fig. 1), indicating that the miRNA sequencing data in this study are representative and will enrich the sugarcane miRNA library. As shown in Fig. 6, the correlation between the sRNA-seq and qRT-PCR experiments was R^2^ = 0.842, which was similar to the report of Bukhari et al. [23] (R^2^ = 0.873). A discrepancy was found between the two detection methods. However, this discrepancy was often observed in previous researches [23, 24], which may probably be due to the different sensitivity and specificity of the two methods [25]. Overall, the results indicated that high-throughput sequencing was a powerful tool for discovering novel and differentially expressed miRNAs.

As reported, plant miRNAs achieve their own functions mainly by regulating the expression of target genes [21]. In this study, among the 68 annotated target genes corresponding to 37 differentially expressed miRNAs, 7 were significantly enriched into mRNA surveillance pathway, selenocompound metabolism, valine, leucine and isoleucine degradation, aminoacyl-tRNA biosynthesis and autophagy pathways (Table 1), suggesting that these genes regulated by the differentially expressed miRNAs may play an important role in the response of sugarcane to SrMV infection. The splicing or translation of target genes guided by miRNAs mainly depends on the complementary degree between miRNA sequence and the corresponding target gene sequence [26]. There is usually a negative regulatory relationship between miRNA and its target gene [26]. In our study, the expression patterns of miR165a-3p, miR395b, miR396a-5p, miR812f, miR1510b-5p, nov_9377, nov_20472 and their target genes were opposite in response to SrMV infection in the two sugarcane varieties (Fig. 7), indicating that these miRNAs may realize their regulatory functions by shearing the target genes. The regulation modes of miR9492, nov_22650 and nov_28432 in the expression level of their target genes in the two sugarcane varieties was different, with a positive regulatory role in ROC22 and a negative regulatory role in FN39 (Fig. 7). This inconsistence has also been observed in the miRNA studies of sugarcane [27] and *Oryza sativa* [28] respectively in response to *Sporisorium scitamineum* and *Rice black-streaked drawf virus* (RBSDV) infections, however the specific regulatory mechanism remains elusive. The expression level of nov_3741 and nov_40875 and their target genes *PRPL2* and *ELP6* were all down-regulated in both two sugarcane varieties (Fig. 7), suggesting that nov_3741 and nov_40875 may positively regulate the expression of *PRPL2* and *ELP6* in sugarcane during SrMV infection.

In plant disease resistance, the speed of the signal recognition, and the effectiveness of defense response in the host after pathogen infection may be the main differences between plant disease-resistant and -susceptible genotypes. In view of this, the plant resistance-related pathways involved in the 15 sugarcane target genes corresponding to 12 candidate differentially expressed miRNAs were analyzed as follows:

### Protein degradation pathway

There are two protein degradation pathways in plants, including the ubiquitin–proteasome system (UPS) or ubiquitin-proteassome pathway (UPP) and plant autophagy [29]. Dantuma et al. [30] showed that viruses can evade the monitoring of the host immune system through the UPP, thus promoting the replication and release of viruses, and blocking or inhibiting the degradation of viral proteins by UPP. In this study, miR396a-5p was predicted to target a deubiquitinase gene *UCH*. In the SMD-susceptible and -resistant varieties infected by SrMV, the expression level of miR396a-5p was increased (3.31- and 1.51-times), while that of the *UCH* gene was inhibited (Fig. 7A), suggesting that miR396a-5p may be involved in *UCH*-mediated protein degradation pathway. Autophagy is highly conserved in eukaryotes and involves in plant immunity against invading pathogens, regulation of plant cell death and antimicrobial defences [29, 31]. ATG8 is a reliable autophagy marker protein and can be used to track the whole process of autophagy from early membrane expansion to final vacuole degradation [32]. Here in our study, nov_20472 was predicted to target *ATG8c*. In both sugarcane susceptible and resistant varieties, the expression level of nov_20472 was increased (2.56- and 1.64-times), while that of the *ATG8c* was decreased (0.53- and 0.48-times) (Fig. 7A). These results suggest that autophagy may be induced by SrMV infection, involving a negative regulatory relationship between nov_20472 and its target gene *ATG8c* in both sugarcane varieties during response. There are various ATG-related genes in autophagy, such as *OsATG8a*-*OsATG8i* in the genome of *O. sativa* [33]. ATG8 orthologs in higher eukaryotes are known to act at different steps of autophagy [34], however the role of ATG8s and autophagy pathway in sugarcane response to SrMV attack needs to be further investigated.

### Plant hormone signal transduction pathway

Plant hormones, such as auxin, jasmonic acid (JA), SA and abscisic acid (ABA), can participate in the regulation of plant growth and development and adversity stress response [35]. In this study, nov_22650 was predicted to target the *ARF6* gene, and their expression level in the two sugarcane varieties infected by SrMV is quite different (Fig. 7C). In ROC22, the expression level of both nov_22650 and *ARF6* was increased (3.38- and 1.52-times). However, the expression level of nov_22650 was decreased (0.41-times) and *ARF6* was increased (2.13-times) in FN39. Auxin/indole-3-acetic acid inducible gene (*AUX/IAA*), a primary auxin-responsive gene, can be rapidly accumulated after auxin stimulation and negatively regulate auxin signal transduction [36]. Padmanabhan et al. [37] found that *Tobacco mosaic virus* (TMV) replicase protein could interact with the *Arabidopsis* AUX/IAA protein and induce specific disease symptoms. Tiwari et al. [38] validated that AUX/IAA protein formed homologous or heterodimers with auxin response factor (ARF), thereby affecting the expression of downstream genes and achieving the auxin stimulus response. These results suggest that nov_22650 may play a negative regulation role in *ARF6* in FN39, but a positive regulation role in *ARF6* in ROC22, which then interferes with the plants’s auxin response system.

ABA is usually considered as a negative regulatory factor in plant disease resistance [39]. Zhou et al. [40] have demonstrated that elongator protein (ELP) plays a key role in ABA response, oxidative stress and anthocyanin synthesis in plants. *ELP*2 gene can rapidly respond to the immune response after *Pst* DC3000 infection in *Arabidopsis* [41]. *ELP6*, another member of the ELP family, was predicted to be targeted by nov_40875. The expression level of nov_40875 and *ELP*6 was all down-regulated in sugarcane susceptible and resistant varieties after SrMV infection, among which the transcript level of nov_40875 was 0.76- and 0.24-times of the control, and that of *ELP6* was 0.54- and 0.61-times of the control (Fig. 7B). It is speculated that nov_40875 may positively regulate *ELP6* expression, which affects sugarcane ABA signal transduction and participates in the immune response of sugarcane to SrMV attack. As reported, nuclear factor Y (NFY) family can respond to ABA stimulation by interacting with bZIP transcription factors [42]. Palmeros-Suárez et al. [43] found that the overexpression of *Amaranthus hypochondriacus AhNFYC* in *Arabidopsis* increased the sensitivity of transgenic seedlings to ABA and affected plant secondary metabolism, growth and development, and gene expression related to ABA stimulation. In this study, miR1510b-5p was predicted to target *NFYC-1*. In sugarcane susceptible and resistant varieties infected by SrMV, the expression level of miR1510b-5p was increased (10.81- and 3.05-times), and that of *NFYC-1* was suppressed (0.62- and 0.52-times) (Fig. 7A). It is thus speculated that the up-regulated expression of miR1510b-5p may negatively regulate the expression level of its target gene *NFYC-1* and interfere with the response of ABA signaling pathway. Based on the above findings, we speculated that the differentially expressed miRNAs reflect the close relationship of the auxin and ABA signal transduction pathways in sugarcane during SrMV infection.

### Thiometabolic pathway

Sulfur can help to respond to various biotic and abiotic stresses in plants [44]. Sulfate transporter (ST) and ATP sulfurylases (ATPs), including low-affinity sulfur transporter (LAST) and high-affinity sulfur transporter (HAST), are the key proteins and enzymes that participate in sulfur transport and absorption [18]. Sanda et al. [45] proved that the sulfite metabolite sulfate is an essential component for the formation of chloroplast membranes. Sulfate-derived β-1,3-glucan sulfate (PS3) can cause immune response under TMV infection [46]. In this study, miR395b was predicted to target both *ATPs-3* and *LAST-3*. After being infected by SrMV, miR395b was up-regulated (1.94-times), and *ATPs-3* and *LAST-3* were down-regulated (0.41- and 0.65- times) in ROC22 (Fig. 7). On the contrary, miR395b was down-regulated (0.55-times), and *ATPs-3* and *LAST-3* were up-regulated (2.52- and 1.39-times) in FN39 (Fig. 7). It is deduced that there may be a negatively regulated mode between miR395b and *ATPs-3* or *LAST-3* gene, which affects the sulfur metabolism pathway and participates in the process of sugarcane response to SrMV attack.

### Plant-pathogen interaction pathway

Calcium ion (Ca^2+^), as the second messenger regulating cell function, is one of the early responses of plants to pathogen infection [47]. CML is a Ca^2+^ binding protein and one of the main Ca^2+^ sensors. Ma et al. [48] found that the innate immune response of *cml24-4* mutants in *Arabidopsis* under *P. syringae* infection was weakened compared with wild type plants. In this study, miR9492 was predicted to target *CML10*. In both sugarcane susceptible (ROC22) and resistant (FN39) varieties infected by SrMV, the expression level of miR9492 was up-regulated (3.16- and 1.97-times), however *CML10* was up-regulated (1.94-times) in ROC22 and down-regulated (0.48-times) in FN39 (Fig. 7A). It is concluded that the regulation of miR9492 on *CML10* which is involved in the Ca^2+^-mediated signaling pathway was opposite in resistant and susceptible sugarcane varieties in response to SrMV infection. PP2A is involved in plant metabolism and programmed cell death [49]. It can dephosphorylate the Bcl-2 protein with anti-apoptotic activity, inhibit the apoptotic activity of Bcl-2 and promote cell apoptosis [50]. As an important component of the PP2A core enzyme, the catalytic subunit PP2Ac forms a stable PP2A holoenzyme together with other PP2A subunits, stably exists in cells and plays a dephosphorylation function [51]. In this study, nov_28432 was predicted to target *PP2Ac*. After being infected by SrMV, the expression level of nov_28432 and *PP2Ac* in ROC22 were up-regulated (2.93- and 1.50-times). In FN39, the expression level of nov_28432 was decreased (0.35-times) and that of *PP2Ac* was increased (1.50-times) (Fig. 7C), suggesting that the down-regulated expression of nov_28432 in resistant sugarcane variety may promote the up-regulated expression of *PP2Ac* and enhance the dephosphorylation of sugarcane in the process of defense response, thus improving the resistance of FN39 to SrMV infection.

### Chloroplast function regulation

Virus infection can destruct the structure and function of the chloroplast in the cell and thus affect plant photosynthesis [52]. The content and activation degree of Ribulose-1, 5-bishosphate carboxylase/oxygenase (Rubisco) are closely related to photosynthetic rate [53]. In higher plants, Rubisco consists of eight large subunits (LSUs) encoded by the chloroplast *rbc*L gene and eight small subunits (SSUs) encoded by a nuclear *Rbc*S gene family [54]. As a molecular chaperone, Rubisco subunit binding protein (rbcBP) can help complete the correct structural assembly of rbc polypeptide [55]. In sugarcane infected by SCSMV, SCSMV P3 protein interacted with Rubisco large subunit to inactivate Rubisco, resulting in chloroplast dysfunction and mosaic disease [56]. In this study, miR165a-3p was predicted to target *rbcLBP*. In ROC22 infected by SrMV, the expression level of miR165a-3p was increased (4.84-times) and that of *rbcLBP* was decreased (0.46-times). Conversely, the expression level of miR165a-3p was decreased (0.72-times) and that of *rbcLBP* was increased (1.59-times) in FN39 (Fig. 7C). It is inferred that the up-regulated expression of miR165a-3p in the susceptible sugarcane variety may negatively regulate the expression level of *rbcLBP*, which then interfere with the normal function of the chloroplast and make ROC22 more prone to mosaic disease than FN39. In addition, chloroplast ribosomes are mainly composed of the 30S and 50S subunits, including 59 plastid ribosomal proteins (PRPs) [57]. In this study, miR812f and nov_3741 were predicted to target *PRPS6* and *PRPL2*, respectively. Among them, the transcripts of nov_3741 and *PRPL2* were decreased in both ROC22 and FN39 infected by SrMV (Fig. 7B), suggesting that *PRPL2* may be positively regulated by nov_3741 and participate in the protein synthesis pathway of sugarcane chlorophyllin. Besides, the expression level of miR812f was significantly up-regulated in the two sugarcane varieties (2.46- and 476.33-times), but its target gene *PRPS6* was slightly down-regulated (0.96- and 0.95-times) (Fig. 7A), indicating that miR812f may positively regulate *PRPS6* expression and affect the chloroplast function.

It is generally recognized that, after pathogen infection, the HR response will be triggered in plants [58]. H_2_O_2_ is an important signaling molecule in plant stress response. SA can affect H_2_O_2_ accumulation and induce plant disease resistance and allergic reaction by regulating the changes of key enzymes in plant defense response [58]. In addition, ET signaling plays an important role in programmed cell death in plants [59]. In this study, the DAB staining colors in tobacco leaves transiently overexpressed sugarcane nov_3741, nov_22650 and nov_40875 were darker than the control leaves (Fig. 8C), indicating that H_2_O_2_ content was accumulated and HR response appeared early. In addition, the overexpression of these three miRNAs induced the expression of immune related marker genes in tobacco (Fig. 8D). Notably, nov_40875 was predicted to target NAC domain-containing protein 78 (*NAC078*), peptide methionine sulfoxide reductase A2-1 (*MSRA2-1*), peptidyl-prolyl cis–trans isomerase CYP20-3 (*CYP20-3*) genes and so on (Fig. 3). NAC proteins are plant specific transcriptional factors that are involved in biotic and abiotic stresses. Transient expression of *Glycine max GmNAC6* gene promoted cell death and hypersensitive-like responses in soybean [60]. *MSRA2-1* is an important antioxidant gene which can participate in oxidative stress [61]. CYPs (cyclophilins) are ubiquitous proteins of the immunophilin superfamily with proposed functions in protein folding, protein degradation, stress response and signal transduction [62]. nov_22650 was predicted to target *ARF6* (Fig. 3). Tian et al. [63] showed that the H_2_O_2_-induced oxidation enhanced BRASSINAZOLE-RESISTANT1 (BZR1)-binding affinity with ARF6 and mediated signaling crosstalks with plant hormone signaling. From all the above, sugarcane nov_3741, nov_22650 and nov_40875 may not only target *PRPL2*, *ARF6* and *ELP6* genes, but also participate in the regulation of chloroplast function, auxin and ABA signaling pathways. Moreover, these three sugarcane miRNAs may function in plant immune response by targeting other mRNAs. Further studies should be conducted to verify the targeting regulatory relations between these sugarcane miRNAs and their target genes in double-luciferase reporter gene assay, stable transgenic plants, Northern and Western blotting, etc.

## Conclusions

**Fig. 9:**
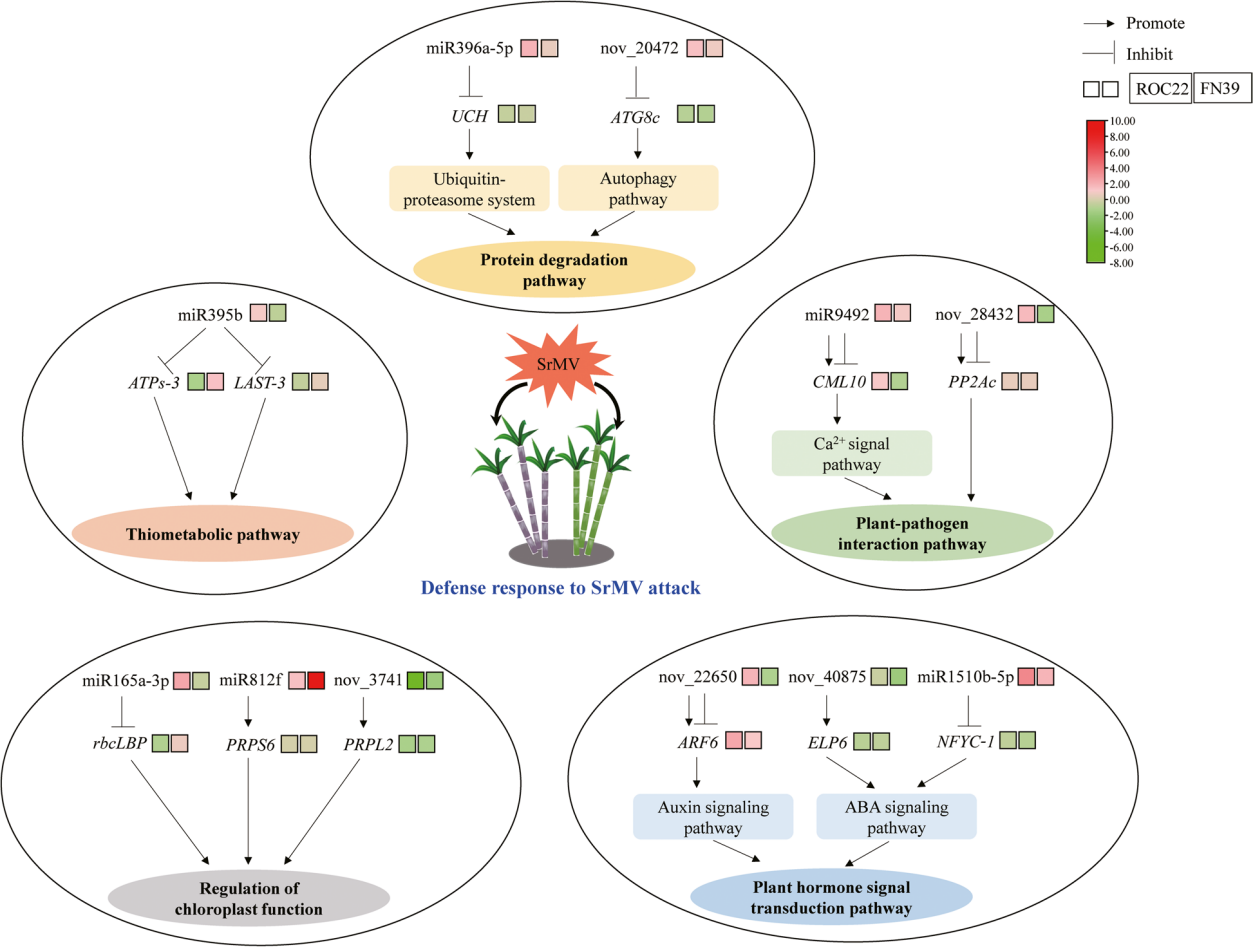
A regulatory network of differentially expressed miRNAs and their predicted target genes in response to *Sorghum mosaic virus* (SrMV) infection in sugarcane. *UCH*, ubiquitin carboxyl-terminal hydrolase; *ATG8c*, autophagy-related protein 8C-like; *ARF6*, auxin response factor 6; *ELP6*, elongator complex protein 6; *NFYC-1*, nuclear transcription factor Y subunit C-1; *ATPs-3*, ATP-sulfurylase 3; *LAST-3*, low affinity sulfate transporter 3-like; *CML10*, calmodulin-like protein 10; *PP2Ac*, phosphatase 2A-3 catalytic subunit-like; *rbcLBP*, Rubisco large subunit-binding protein; *PRPS6*, 30S chloroplastic ribosomal protein S6; and *PRPL2*, 50S chloroplastic ribosomal protein L2. The color code is constructed by TBtools, with the transcript level of the miRNAs and their corresponding target genes transformed as log_2_ (qRT-PCR), ranging from red (up-regulated) to green (down-regulated)

In the present study, a database of miRNAs for sugarcane response to SrMV infection was constructed. A total of 132 mature miRNAs were identified and 1,037 target genes were predicted. After being infected by SrMV, the number of differentially expressed miRNAs in SMD-resistant variety FN39 (30) was more than that in SMD-susceptible variety ROC22 (19). KEGG enrichment analysis showed that these target genes corresponding to differentially expressed miRNAs mainly involved in disease resistance-related pathways, such as mRNA surveillance, plant pathway interaction, sulfur metabolism, and autophagy regulation. A proposed regulatory network of differentially expressed miRNAs and their target genes in sugarcane in response to SrMV infection was drawn (Fig. 9). Furthermore, the transient overexpression showed the involvement of three candidate sugarcane novel miRNAs (nov_3741, nov_22650 and nov_40875) in plant immune responses on tobacco leaves. These results will enrich the sugarcane miRNA library and provide disease resistance genes for breeding sugarcane varieties resistant to mosaic disease by genetic engineering.

## Methods

### Plant materials and treatment

The SMD-susceptible sugarcane variety ROC22 and -resistant variety FN39 [64] were provided by the Key Laboratory of Sugarcane Biology and Genetic Breeding, Ministry of Agriculture and Rural Affairs (Fuzhou, China). Referring to the method of Ling et al. [16], the youngest fully expanded leaf viz + 1 leaf with a visible dewlap (the collar between the leaf blade and sheath) of the nine-month-old sugarcane healthy plants and the plants with mosaic symptoms of these two varieties were selected in the field. After detection by reverse transcription-PCR (RT-PCR) and PCR, the sugarcane plants without SCMV, SrMV, SCSMV, *Suagracne yellow leaf virus* (ScYLV) and *Sugarcane bacilliform virus* (SCBV) were used as the control group, and the plants only infected by SrMV were used as the treatment group. Then the stems of the control and treatment groups were cut into single-bud stem sections, and cultivated in the greenhouse. At 6-to-8 leaf stage, the + 1 and + 2 leaves of virus-free (control group) and SrMV-infected plants with mosaic symptoms (treatment group) were harvested. Each sample contained three biological replicates, and each biological replicate contained three plants. All the collected samples were tested by RT-PCR to verify the presence or absence of SrMV [65].

### Total RNA extraction, small RNA library construction and sequencing

For ROC22, the samples of control groups were labeled as S1–S3, and those of the treatment groups were labeled as S4–S6. For FN39, the samples of control groups were marked as S7–S9, and those of the treatment groups were marked as S10–S12. Total RNA was extracted from the above 12 samples using Trizol reagent (Invitrogen, Shanghai, China). Nanodrop, Qubit 2.0 and Agilent 2100 bioanalyzer were used to detect the purity, concentration and integrity of RNA samples. Before cDNA synthesis, the genomic DNA was removed according to the instructions of the RNase-Free DNase I kit (Promega, Fitchburg, WI, USA). Then the RNA was reversed into the first strand of cDNA referred to the instructions of Prime-Script™ RT Reagent Kit (TaKaRa, Dalian, China). The sequencing (HiSeq2500) and library construction of sugarcane small RNAs were commissioned by Biomarker Biotechnology Co., Ltd. (Beijing, China). The read length was 50 nt.

### Sequencing data processing and analysis

After sRNA-seq, low quality reads, unrecognized reads with an unknown base content ≥ 10%, reads without 3' linker sequences or insert fragments, reads containing ploy A/T/C/G, and the length of reads ˂ 18 nt or > 30 nt were removed. Bowtie software [66] was used to compare the obtained clean reads with the database of Silva (https://www.arb-silva.de/), Rfam (http://rfam.xfam.org/), GtRNAdb (http://lowelab.ucsc.edu/GtRNAdb/) and Repbase (http://www.girinst.org/repbase/), and non-coding RNA (ncRNA) and Repbase were filtered. Then the unannotated reads which contained miRNAs were obtained. Due to the limited number of sugarcane miRNAs in the miRBase database, the filtered reads with length of 18–30 nt were compared with the pre-miRNA and mature miRNA sequences in the public miRBase database (http://www.mirbase.org/) by miRDeep2 software [67] to identify the known miRNAs (conservative miRNAs). The unaligned sequences were filtered and extended to predict the miRNA structure and analyze the novel miRNAs (nonconservative miRNAs). Then the length distribution, base preference, and family members of the identified miRNAs were counted. In addition, the BLAST tool (https://blast.ncbi.nlm.nih.gov/Blast.cgi) was used to compare all miRNAs with the SrMV genome (GenBank accession number: NC_004035.1).

### Analysis of differentially expressed miRNAs

In order to eliminate the influence of the difference in sequencing amount between different samples, the miRNA expression quantity in each sample was normalized using the calculation formula of transcripts per million (TPM) [68]. Then the expression pattern of miRNA in the sample was analyzed. The logarithms of TPM corresponding to the sample and the probability density were respectively represented by abscissa and ordinate, and the overall distribution map of miRNA expression was drawn. The differentially expressed miRNAs between the treatment and control groups were screened by DESeq software [69] with the criteria of |log_2_ (fold change, FC)|≥ 1 and false discovery rate (FDR) ≤ 0.05.

### Prediction and functional annotation of miRNA target genes

Based on our previous transcriptome data of sugarcane [16, 17], the target genes of the identified miRNAs were predicted by TargetFinder v1.6 software [70]. When the score was less than or equal to 3, the transcript was considered as the target gene. BLAST software (https://blast.ncbi.nlm.nih.gov/Blast.cgi) was used to compare the predicted target gene sequence with the database of NR (ftp://ftp.ncbi.nih.gov/blast/db/), Swiss-Prot (http://www.uniprot.org/), GO (http://www.geneontology.org/), COG (http://www.ncbi.nlm.nih.gov/COG/), KEGG (http://www.genome.jp/kegg/), KOG (http://www.ncbi.nlm.nih.gov/KOG/) and Pfam (http://pfam.xfam.org/) to obtain the annotation information of the predicted target genes. And the predicted target genes were subjected to GO (http://www.geneontology.org/) enrichment and KEGG (http://www.genome.jp/kegg/) pathway [20] analyses. Networks between the differentially expressed miRNAs and their corresponding target genes were constructed by Cytoscape software (v3.9.0).

### Cluster analysis of differentially expressed miRNAs

Based on the log_10_ (TPM + 1) values of the 37 identified differentially expressed miRNAs in 12 sRNA-seq samples, the miRNAs with similar expression patterns were clustered by using R package Heatmap version 1.0. Then, Go enrichment analysis of the predicted target genes of these miRNAs (Table S5) with high and low expression abundances was performed, respectively.

### qRT-PCR analysis of miRNAs and their target genes expression

Referring to the sampling method of sRNA-seq, the + 1 and + 2 leaves of virus-free (control group) and SrMV-infected plants with mosaic symptoms (treatment group) were used for expression analysis of miRNAs and their corresponding target genes. For the detection of miRNA expression, the leaf RNA was reversed into cDNA with the RT primers of the selected miRNAs (Table S6) according to the instructions of TaqMan MicroRNA Reverse Transcription Kit (Applied Biosystems, Foster, CA, USA). The cDNA used to detect the expression level of predicted target gene was synthesized by Prime-Script™ RT Reagent Kit (TaKaRa, Dalian, China). qRT-PCR primers of 12 randomly selected differentially expressed miRNAs were designed according to the method of Varkonyi-Gasic et al. [71] (Table S6). miR159 was used as an internal reference gene [13], and the expression of miRNAs was detected by stem-loop method [72]. The correlation of qRT-PCR (log_2_ scale) and sequencing data of the miRNAs was analyzed by scatter plot. Based on the sequences of 15 predicted target genes corresponding to the 12 miRNAs, NCBI Primer designing tool (https://www.ncbi.nlm.nih.gov/tools/primer-blast/) was applied to design their qRT-PCR primers (Table S7). The *PP2A* gene was served as an internal reference gene [13]. ABI 7500 Fast Real-time PCR amplification system (Applied Biosystems, Foster, CA, USA) was used for qRT-PCR analysis of miRNAs and their corresponding target genes. The qRT-PCR system (20 μL) contained 10 μL SYBR Primix Ex Taq™ (2 ×), 0.8 μL 10 μmol/L forward/reverse primer, 2.0 μL cDNA and 6.4 μL ddH_2_O. The qRT-PCR program was 50 °C 2 min, 95 °C 10 min, 95 °C 15 s and 60 °C 1 min for 40 cycles. Three biological and technical replicates were set for each sample, and sterile ddH_2_O was used as the blank control. The qRT-PCR data was calculated by the 2^−ΔΔCt^ algorithm [73], and DPS software was used to analyze the significant difference of the data (*p*-value < 0.05).

### Cloning and transient expression analysis of candidate sugarcane miRNAs

According to the precursor sequences of three candidate novel miRNAs, nov_3741, nov_22650 and nov_40875, their cloning primers were designed using the NCBI Primer designing tool (Table S8). The cDNA of ROC22 leaf infected by SrMV was synthesized by Prime-Script™ RT Reagent Kit (TaKaRa, Dalian, China) and used for the miRNA cloning. The PCR reaction system (25 μL) contained 2.5 μL 10 × Ex Taq buffer, 2.0 μL 10 mmol/L dNTPs, 1.0 μL 20 μmol/L forward/reverse primer, 0.125 μL Ex Taq enzyme (TaKaRa, Dalian, China), 1.0 μL cDNA template and 17.375 μL ddH_2_O. The PCR program was 94 °C 4 min, (94 °C 30 s, 55 °C 30 s, 72 °C 30 s) 35 cycles, 72 °C 10 min. The PCR products were verified by electrophoresis, purification, cloning and sequencing.

Primers of nov_3741, nov_22650 and nov_40875 (Table S8) for constructing the plant overexpression vectors, and the three plasmids of the cloned pre-miRNAs were used for PCR amplification. Then the purified PCR products and the plasmids of plant overexpression vector pCAMBIA 1301 were digested with the enzymes *Bam*H I and *Spe* I and ligated with T4 ligase to construct the recombinant plasmids. The positive plasmids verified by double enzyme digestion were transferred into *Agrobacterium* GV3101 cells (*35::3741*, *35::22650* and *35::40875*), and the *Agrobacterium* contained the empty vector pCAMBIA 1301 was used as the control (*35::00*). According to the transient overexpression method [74], the *Agrobacterium* solution with a concentration of OD_600_ = 0.8 (plus 200 μmol/L acetosyringone) in MS liquid medium was injected into 8-leaf-stage *N. benthamiana* leaves. The agroinfiltrated plants were cultivated at 24 °C for 2 days for DAB staining and expression level analysis of the target genes and nine tobacco immune-related marker genes [74]. The *N. benthamiana* immune-related marker genes were hypersensitive response marker genes *HSR201*, *HSR203* and *HSR515*, SA-responsive genes *NPR1*, *PR-1a/c*, *PR2* and *PR3*, and ET synthesis-depended genes *EFE26* and *Accdeaminase* (Table S8). *EF1-α* was used to normalize the transcript level (Table S8). The qRT-PCR system, program and data statistics referred to the above method of qRT-PCR analysis of target genes expression.

## Supplementary Information

**Additional file 1: Figure S1.** The targeting sites of the selected 12 miRNAs and their 15 target genes predicted by TargetFinder v1.6 software. When the score of miRNA that matches the mRNA is less than or equal to 3, the transcript sequence is considered as the miRNA target gene. Range represents the interval of mRNA used for alignment. Black lines indicate matched RNA base pairs, and two dots show a GU mismatch whereas none dot represents other types of mismatch. The gene ID follows the name of miRNAs and their target genes. **Figure S2.** The original image of PCR products of the three candidate sugarcane pre-miRNAs detected by electrophoresis. M, DNA marker 15,000 + 2,000 bp; 1, pre-nov_3741; 2, pre-nov_22650; and 3, pre_nov_40875.

**Additional file 2: Table S1.** The statistic result of sRNA-seq data in 12 samples. **Table S2.** The statistic result of annotation and classification of the small RNAs (%). **Table S3.** The differentially expressed miRNAs in sugarcane varieties ROC22 and FN39 infected by SrMV. **Table S4.** List of 653 annotated predicted target genes corresponding to 132 identified miRNAs. **Table S5. **List of predicted target genes corresponding to the differentially expressed miRNAs. **Table S6.** The quantitative primers ofthe 12 selected miRNAs. **Table S7. **The quantitative primers of the 15 predicted target genes. **Table S8.** Primers used for cloning the candidate miRNAs and expression analysis of the *Nicotiana benthamiana* immune-related marker genes.

## Data Availability

The original contributions presented in the study are included in the article/Supplementary Material, further inquiries can be directed to the corresponding authors. The sequencing reads are deposited in the National Center for Biotechnology Information under the BioProject number PRJNA793396.

## Abbreviations

SMD: Sugarcane Mosaic Disease
SCMV: Sugarcane Mosaic Virus
SrMV: Sorghum Mosaic Virus
SCSMV: Sugarcane Streak Mosaic Virus
miRNA: MicroRNA
sRNA: Small RNA
TuMV: Turnip Mosaic Virus
TYMV: Tymovirus
ACMV: African Cassava Mosaic Virus
PP2A: Protein Phosphatase 2A
sRNA-seq: Small RNA Sequencing
qRT-PCR: Quantitative Real-Time PCR
rRNA: Ribosomal RNA
snoRNA: Small Nucleolar RNA
tRNA: Transfer RNA
Repbase: Repeat Sequence
NR: Non-Redundant Protein Sequence Database
GO: Gene Ontology
COG: Clusters of Orthologous Groups
KEGG: Kyoto Encyclopedia of Genes and Genomes
KOG: EuKaryotic Orthologous Groups
ATPs-3: ATP-Sulfurylase 3
ATG8c: Autophagy-Related Protein 8C-Like
LAST-3: Low Affinity Sulfate Transporter 3-Like
CML: Calmidulin-Like Protein
UCH: Ubiquitin Carboxyl-Terminal Hydrolase
PRPS6: 30S Plastid Ribosomal Protein S6
NFYC-1: Nuclear Transcription Factor Y Subunit C-1
PRR73: Pseudo-Response Regulator 73
PRPL2: 50S Chloroplastic Ribosomal Protein L2
ELP6: Elongator Complex Protein 6
rbcLBP: Rubisco Large Subunit-Binding Protein
ARF6: Auxin Response Factor 6
PP2Ac: Phosphatase 2A-3 Catalytic Subunit-Like
H_2_O_2_: Hydrogen Peroxide
DAB: 3,3'-Diaminobenzidine Solution
HR: Hypersensitive Response
SA: Salicylic Acid
ET: Ethylene
RBSDV: Rice Black-Streaked Drawf Virus
UPS: Ubiquitin-Proteasome System
UPP: Ubiquitin-Proteassome Pathway
JA: Jasmonic Acid
ABA: Abscisic Acid
AUX/IAA: Auxin/Indole-3-Acetic Acid Inducible Gene
ARF: Auxin Response Factor
TMV: Tobacco Mosaic Virus
ELP: Elongator Protein
NFY: Nuclear Factor Y
ST: Sulfate Transporter
ATPs: ATP Sulfurylases
LAST: Low-Affinity Sulfur Transporter
HAST: High-Affinity Sulfur Transporter
PS3: Sulfate-Derived β-1,3-Glucan Sulfate
Ca^2+^: Calcium Ion
Rubisco: Ribulose-1, 5-Bishosphate Carboxylase/Oxygenase
LSUs: Rubisco Consists of Eight Large Subunits
SSUs: Small Subunits
rbcBP: Rubisco Subunit Binding Protein
PRPs: Plastid Ribosomal Proteins
MSRA2-1: Peptide Methionine Sulfoxide Reductase A2-1
CYP20-3: Peptidyl-Prolyl Cis–Trans Isomerase CYP20-3
NAC078,: NAC Domain-Containing Protein 78
RT-PCR: Reverse Transcription-PCR
ScYLV: Suagracne Yellow Leaf Virus
SCBV: Sugarcane Bacilliform Virus
ncRNA: Non-Coding RNA
TPM: Transcripts Per Million
FC: Fold Change
FDR: False Discovery Rate
