# Supplementary material for: Systematic identification of miRNA-regulatory networks unveils their potential roles in sugarcane response to Sorghum mosaic virus infection
Source: BMC Plant Biol. 2022 May 19;22:247. doi: 10.1186/s12870-022-03641-6 (PMC9118776; doi:10.1186/s12870-022-03641-6)
Supplement: Supplementary file 1 — Additional file 1: Figure S1. The targeting sites of the selected 12 miRNAs and their 15 target genes predicted by TargetFinder v1.6 software. When the score of miRNA that matches the mRNA is less than or equal to 3, the transcript sequence is considered as the miRNA target gene. Range represents the interval of mRNA used for alignment. Black lines indicate matched RNA base pairs, and two dots show a GU mismatch whereas none dot represents other types of mismatch. The gene ID follows the name of miRNAs and their target genes. Figure S2. The original image of PCR products of the three candidate sugarcane pre-miRNAs detected by electrophoresis. M, DNA marker 15,000 + 2,000 bp; 1, pre-nov_3741; 2, pre-nov_22650; and 3, pre_nov_40875. [file 12870_2022_3641_MOESM1_ESM.pdf]

# Systematic identification of miRNA-regulatory networks unveils their potential roles in sugarcane response to *Sorghum mosaic virus* infection

**Yachun Su**[1]

**E-mail:** syc2009mail@163.com

**Qiong Peng**[2]

**E-mail:** pengqiongfj@163.com

**Hui Ling**[3]

**E-mail:** linghuich@163.com

**Chuihuai You**[4]

**E-mail:** you123chui@163.com

**Qibin Wu**[1]

**E-mail:** wqbaidqq@163.com

**Liping Xu**[1,*]

**E-mail:** xlpmail@126.com

**Youxiong Que**[1,*]

**E-mail:** queyouxiong@126.com

[1] Key Laboratory of Sugarcane Biology and Genetic Breeding, Ministry of Agriculture and Rural Affairs, Fujian Agriculture and Forestry University, Fuzhou 350002, Fujian, China

[2] Fuzhou Institute of Agricultural Sciences, Fuzhou 350018, Fujian, China

[3] College of Agriculture, Yulin Normal University, Yulin 537000, Guangxi, China

[4] College of Life Sciences, Fujian Agriculture and Forestry University, Fuzhou 350002, Fujian, China

***Correspondence should be addressed to xlpmail@126.com, queyouxiong@126.com**

**The full postal address of the submitting author Youxiong Que is as follows:** Key Laboratory of Sugarcane Biology and Genetic Breeding, Ministry of Agriculture, Fujian Agriculture and Forestry University, Fuzhou 350002, China

## Supplementary Figures

(1) score=3, range=1257-1278

```
3’ UUCAAGUU-CUUUCGACACCUU 5’ miR396a-5p (conservative_CL13043Contig1_12201)
   |||| ||| |||||||||||:|
5’ AAGUCCAAUGAAAGCUGUGGGA 3’ UCH (CL5337Contig1)
```

(2) score=3, range=76-96

```
3’ UUCAAGUUCUUUCGACACCUU 5’ miR396a-5p ( conservative_CL13043Contig1_12201)
   |||   |:||||||||||||:
5’ AAGGCCGAGAAAGCUGUGGAG 3’ RDDP (Sugarcane_Unigene_BMK.42384)
```

(3) score=3, range=642-663

```
3’ UUCAAGUUCUUUC-GACACCUU 5’ miR396a-5p (conservative_CL13043Contig1_12201)
   | ||||||||||| ||||||||
5’ ACGUUCAAGAAAGCCUGUGGAA 3’ GRF2 ( gi34938144)
```

(4) score=0, range=3306-3326

```
3’ UCGGAACCGUAGUAGUAGGCG 5’ miR812f (conservative_CL4784Contig1_6602)
   |||||||||||||||||||||
5’ AGCCUUGGCAUCAUCAUCCGC 3’ PRPS6 (CL4784Contig1)
```

(5) score=3, range=1146-1166

```
3’ GCAUAAGUGGAGUUAGGUACA 5’ miR1510b-5p (conservative_T1_Unigene_BMK.40479_34605)
   ||||||||||||||:||  ||
5’ CGUAUUCACCUCAAUUCACGU 3’ NFYC-1 (Sugarcane_Unigene_BMK.56105)
```

(6) score=3, range=514-537

```
3’ UAGACCUACAAAGUUUCAUCUAG 5’ miR9492 (conservative_CL26672Contig1_15829)
   :|:|||||||||||||| ||||||
5’ GUUUGGAUGUUUCAAAACUAGAUC 3’ CML10 (gi35101728)
```

(7) score=3, range=2754-2772

```
3’ CGAAGUCCUUUCUACUGUGG 5’ nov_9377 (unconservative_CL9044Contig1_9377)
   | |||| ||||||||||||
5’ GGUUCA-GAAAGAUGACACG 3’ PRR73 (T3_Unigene_BMK.50033)
```

(8) score=2, range=111-132

```
3’ ACGAUGUCAUG-AAAAAAAUGU 5’ nov_20472 (unconservative_Sugarcane_Unigene_BMK.24423_20472)
   |||||||||| |||||||||||
5’ UGCUACAGUACUUUUUUUUACA 3’ ATG8c (CL7998Contig1)
```

(9) score=0, range=1953-1972

```
3’ AGGUGAACCGAUGUAGGCGG 5’ nov_3741 (unconservative_CL4778Contig1_3741)
   ||||||||||||||||||||
5’ UCCACUUGGCUACAUCCGCC 3’ PRPL2 (CL4778Contig1)
```

(10) score=1. 5, range=20-43

```
3’ UUGUCAUAAAAGUCGGUACUGAAA 5’ nov_40875 (unconservative_T3_Unigene_BMK.49787_40875)
   |:||||| ||||||||||||||||
5’ AGCAGUACUUUCAGCCAUGACUUU 3’ ELP6 (Sugarcane_Unigene_BMK.46721)
```

(11) score=2. 5, range=363-379

```
3’ CUUACUUCGGACCAGGCU 5’ miR165a-3p (conservative_Sugarcane_Unigene_BMK.26076_20678)
   ||:||||| ||||||||
5’ GAGUGAAG-CUGGUCCGA 3’ rbcLBP (Sugarcane_Unigene_BMK.68061)
```

(12) score=1, range=577-596

```
3’ CUCAAGGGGGUUUGUGAAGU 5’ miR395b (conservative_Sugarcane_Unigene_BMK.11814_18991)
   |||||||:||||||||||||
5’ GAGUUCCUCCAAACACUUCA 3’ ATPs-3 (Sugarcane_Unigene_BMK.53035)
```

(13) score=0, range=328-347

```
3’ CUCAAGGGGGUUUGUGAAGU 5’ miR395b (conservative_Sugarcane_Unigene_BMK.11814_18991)
   ||||||||||||||||||||
5’ GAGUUCCCCCAAACACUUCA 3’ LAST-3 (CL26973Contig1)
```

(14) score=3, range=342-362

```
3’ UUUUUGAUACCUAGAUUUUUU 5’ nov_28432 (unconservative_Sugarcane_Unigene_BMK.66937_28432)
   |||||||||| |||||:||||
5’ AAAAACUAUGUAUCUAGAAAA 3’ PP2Ac (CL9Contig5)
```

(15) score=3, range=125-142

```
3’ CAAGCAGAUUAAUACGGCA 5’ nov_22650 (unconservative_Sugarcane_Unigene_BMK.39299_22650)
   |||| |||||||||||:|
5’ GUUC-UCUAAUUAUGCUGA 3’ ARF6 (CL26Contig3)
```

**Figure S1** The targeting sites of the selected 12 miRNAs and their 15 target genes predicted by TargetFinder v1.6 software. When the score of miRNA that matches the mRNA is less than or equal to 3, the transcript sequence is considered as the miRNA target gene. Range represents the interval of mRNA used for alignment. Black lines indicate matched RNA base pairs, and two dots show a GU mismatch whereas none dot represents other types of mismatch. The gene ID follows the name of miRNAs and their target genes.

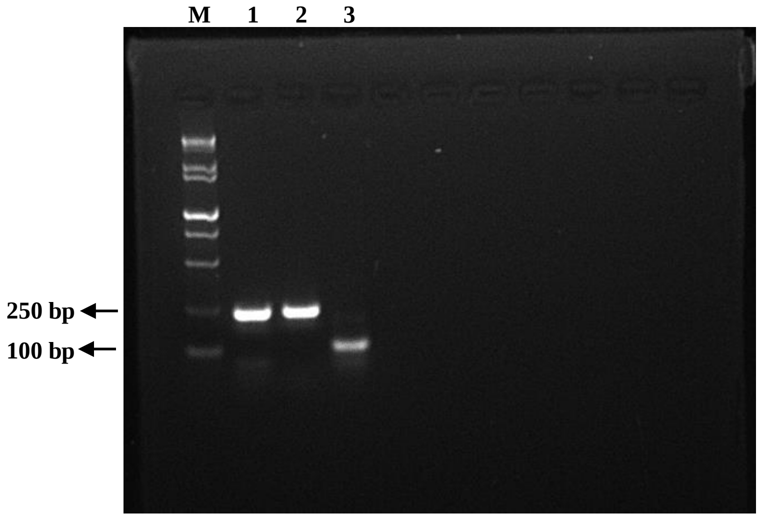


**Figure S2** The original image of PCR products of the three candidate sugarcane pre-miRNAs detected by electrophoresis. M, DNA marker 15,000 + 2,000 bp; 1, pre-nov_3741; 2, pre-nov_22650; and 3, pre_nov_40875.
